# Antimicrobial Effects of Lactoferrin against *Helicobacter pylori* Infection

**DOI:** 10.3390/pathogens12040599

**Published:** 2023-04-14

**Authors:** Ichiro Imoto, Taro Yasuma, Corina N. D’Alessandro-Gabazza, Satoko Oka, Moriharu Misaki, Noriyuki Horiki, Esteban C. Gabazza

**Affiliations:** 1 Digestive Endoscopy Center, Doshinkai Tohyama Hospital, Minami-shinmachi 17-22, Tsu, Mie 514-0043, Japan; md-imoto@toyama-hp.or.jp; 2 Department of Immunology, Mie University Faculty and Graduate School of Medicine, Edobashi 2-174, Tsu, Mie 514-8507, Japan; t-yasuma0630@clin.medic.mie-u.ac.jp (T.Y.);; 3 Department of Internal Medicine, Doshinkai Tohyama Hospital, Minami-shinmachi 17-22, Tsu, Mie 514-0043, Japan; 4 Digestive Center, Mie University Hospital, Edobashi 2-174, Tsu, Mie 514-8507, Japan

**Keywords:** *Helicobacter pylori*, lactoferrin antimicrobial effect, eradication

## Abstract

*Helicobacter* (*H.*) *pylori* is the primary causative agent of various gastroduodenal diseases. *H. pylori* is an adapted microorganism that has evolved to survive in the acidic conditions of the human stomach, possessing a natural strategy for colonizing harsh environments. Despite the implementation of various eradication regimens worldwide, the eradication rate of *H. pylori* has decreased to less than 80% in recent years due to the emergence of antibiotic-resistant strains. This has posed a significant challenge in treating *H. pylori* infection, as antibiotic resistance and side effects have become increasingly problematic. Lactoferrin, a member of the transferrin family, is an iron-binding protein with antioxidant, antibacterial, antiviral, and anti-inflammatory properties that promote human health. The concentrations of lactoferrin in the gastric juice and mucosa significantly increase during *H. pylori* infection and are strongly correlated with the severity of gastric mucosal inflammation. Numerous researchers have studied the antimicrobial properties of lactoferrin both in vitro and in vivo. In addition, recent studies have investigated the addition of oral lactoferrin supplementation to *H. pylori* eradication therapy, even though monotherapy with lactoferrin does not eradicate the microorganism. In this article, we reviewed the survival strategy of *H. pylori* to evade the antimicrobial activity of human lactoferrin and explore the potential of lactoferrin in *H. pylori* eradication therapy.

## 1. Introduction

*Helicobacter* (*H.*) *pylori* is the primary causative agent of gastritis, peptic ulcer diseases, gastric cancer, and mucosa-associated lymphoid tissue (MALT) lymphoma [1]. Other causes of gastric pathology include drugs, alcohol, or stress (e.g., trauma, burn). *H. pylori* eradication therapy is widely used due to its beneficial effects such as reduction of gastric mucosal inflammation, prevention of peptic ulcer recurrence, decreased risk of developing gastric cancer, and reduced prevalence and treatment cost of dyspepsia [2,3,4,5,6,7]. Several guidelines on *H. pylori* treatment have been proposed worldwide [8,9,10]. Triple therapy combining a proton pump inhibitor (PPI) with two antimicrobial agents is recommended as the standard first-line treatment for *H. pylori* [11,12]. In recent years, however, the eradication rate of *H. pylori* has decreased to less than 80% due to the increased prevalence of resistant strains. The standard therapy with antibiotics typically includes amoxicillin (AMPC), clarithromycin (CAM), metronidazole (MET), and levofloxacin (LVFX); however, the emergence of resistant strains to CAM, MET, and LVFX has become a major concern [13,14]. Therefore, therapeutic guidelines from European countries have recommended different eradication regimens for regions with CAM resistance rates above and below 15% [8,9,10]. In Japan, the use of a first-line triple therapy consisting of vonoprazan (a potassium-competitive acid blocker), AMPC, and CAM has resulted in an impressive 92.6% eradication rate in just 7 days [15]. The vonoprazan-based triple therapy has demonstrated a significantly higher eradication rate of 82.0% for CAM-resistant strains, compared to the 40.0% eradication rate for lansoprazole-based triple therapy [15,16]. Unfortunately, vonoprazan is not available in all countries or provinces. Therefore, it is recommended that patients in areas with high rates of CAM resistance and no access to vonoprazan receive bismuth-based quadruple therapy or non-bismuth concomitant quadruple therapy for 10–14 days as the first-line treatment [8,9,10]. Despite the improved eradication rate of *H. pylori* achieved by the regimens above, the emergence of resistant strains and the side effects of these agents have become a major concern [17]. Therefore, it is desirable to use other safer agents in combination. In addition, dysbiosis of the stomach and intestinal microbiota and increased incidence of other chronic diseases (e.g., allergic disease, Barrett’s esophagus) have also been reported after eradication therapy of *H. pylori* [6,18,19]. Based on this, evaluating the benefit–risk ratio for each patient has been recommended before indicating *H. pylori* eradication therapy [19,20].

In 1940, Sorensen et al. were the first to identify and isolate lactoferrin (LF), an iron-binding glycoprotein, from bovine milk [21]. The concentration of LF in human colostrum ranges from 5 to 8 mg/mL, while in mature milk, it is 1–3 mg/dl [22,23]. The high concentrations of LF in colostrum are thought to play an important role in infant development and infection prevention. LF is present in exocrine fluids such as lacrimal fluid and saliva [24] and the second granules of polymorphonuclear leukocytes [25] and has a variety of effects, including growth inhibition of various microorganisms, immunomodulation, anti-inflammatory effects, and cancer prevention [24,26]. Despite histological evidence of LF expression in the gastric mucosa, the significance of LF during *H. pylori* infection has remained uncertain [27]. For the first time, we reported that concentrations of LF in the gastric juice and mucosa significantly increased during *H. pylori* infection and are closely correlated with the degree of gastric mucosal inflammation [28,29]. However, the low concentration of LF in vivo makes it uncertain whether it has an antimicrobial effect against *H. pylori*. Therefore, we investigated the antimicrobial effect of LF on *H. pylori* in vitro and in vivo [30,31,32]. In this article, we review the antimicrobial impact of LF against *H. pylori* and discuss its potential therapeutic value in *H. pylori* eradication therapy.

## 2. Protective Activity of Lactoferrin

LF plays an important role in iron homeostasis [24]. It binds to and promotes the absorption and transport of iron in the gastrointestinal tract [26]. LF may suppress bacteria growth by depriving them of iron and exerts bactericidal activity by enhancing the permeability of the bacterial membrane [33]. In addition, LF degradation by pepsin releases lactoferricin (LFcin), another potent antibacterial peptide [32,34]. Interestingly, LFcin was reported to inhibit the urease activity of *H. pylori* [32]. The production of ammonia by urease released by *H. pylori* is a critical factor that allows the bacterium’s survival in the stomach’s acid environment [35]. Reports also support the antiviral effects of LF. It may inhibit viral penetration into host cells by binding to cell surface proteoglycans, binding to viral proteins, or interfering with intracellular viral transport [26,33]. In addition to the direct effect of LF on *H. pylori*, its anti-inflammatory activity may also explain the therapeutic properties of LF in *H. pylori*-associated pathology including gastric injury [36,37,38,39]. LF may also modulate the inflammatory response by interacting with immune cell surface receptors, regulating intracellular signal pathways, and controlling the production of inflammatory cytokines and the oxidant activity of iron [40,41,42,43]. In addition, evidence suggests that LF may exhibit anticancer activity by inhibiting the migration and proliferation and inducing apoptosis of cancer cells [26,44,45].

## 3. Intragastric Lactoferrin and *H. pylori* Colonization

Most living organisms require iron for survival. Iron has low solubility; therefore, in mammals, it is typically bound to hemoglobin, an oxygen carrier, and to proteins such as intracellular ferritin for storage, extracellular transferrin for iron transport to cells, and LF to reduce iron availability for microorganisms [46,47]. The expression of LF has been described in the fundic and pyloric glands of the gastric mucosa, particularly, in the inflamed mucosa; yet its physiological role in the stomach remains unknown [27]. Nakao et al. demonstrated that increased levels of LF in the gastric juice and gastric mucosa were strongly associated with *H. pylori* infection [28,29].

**Figure 1:**
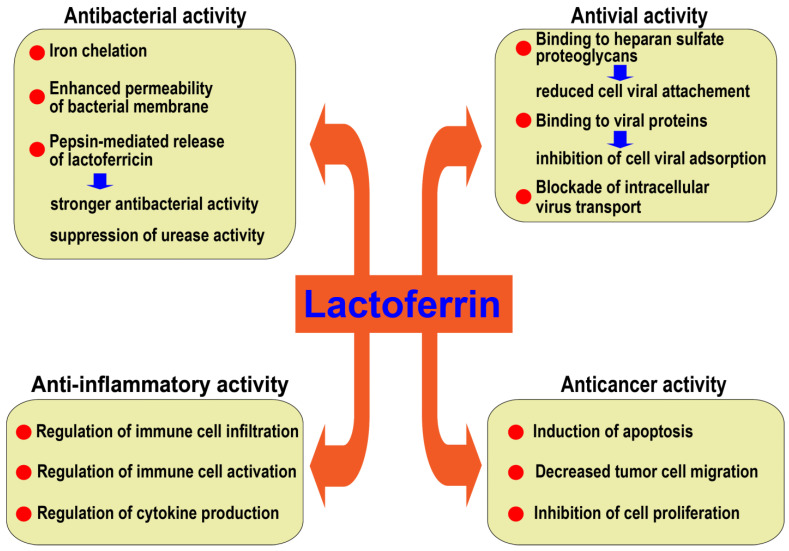
The beneficial effects of lactoferrin. Lactoferrin is protective in the stomach by several mechanisms including anti-inflammatory, antimicrobial, and anti-cancer activities.

Lu et al.’s subsequent report showed that the increased levels of LF in gastric tissue were caused by *H. pylori* colonization, as demonstrated by the Mongolian gerbil’s model [48]. The Mongolian gerbil is a rodent model that recapitulates many features of *H. pylori*-induced gastric disease in humans [49]. In addition, it is widely recognized that the primary antimicrobial action of LF is to deprive bacteria of iron, thus inhibiting their growth [50]. However, since pepsin, an acidic enzyme, degrades LF, it is conceivable that the antimicrobial activity of LF is weakened by pepsin degradation in the stomach’s acidic environment [51]. However, the resulting degradation product, lactoferricin, exhibits an even stronger antimicrobial activity than lactoferrin (Figure 1) [32].

*H. pylori* is a microorganism adapted to survive in the acidic environment of the human stomach, possessing urease activity. *H. pylori* has developed a survival strategy to colonize hostile environments, such as the human stomach, leading to chronic, persistent infections [35]. Therefore, it is conjectured that *H. pylori* has an iron acquisition system for survival in the stomach, enabling it to circumvent the antimicrobial activity of human LF (h-LF). In contrast to other mucosal colonizers with siderophore-mediated iron uptake mechanisms, *H. pylori* has not been demonstrated to synthesize siderophores [52]. Gastric acid facilitates the release of iron from ingested food, yet *H. pylori* does not inhabit the acidic environment of the gastric lumen, instead preferring to colonize the neutral environment of the epithelial cell surface and the overlying mucus layer [53]. Iron within the gastric mucus layer is bound to lactoferrin (LF) or other glycoproteins [54]. *H. pylori* is unable to circumvent the capacity of partially saturated (apo) lactoferrin (LF) in iron acquisition [55]. However, *H. pylori* can exploit iron from fully saturated (holo) transferrin, the predominant form endocytosed by epithelial cells [56]. Additionally, *H. pylori* can utilize human lactoferrin (h-LF) but not bovine lactoferrin (b-LF) as an iron source in an iron-restricted medium and expresses the lactoferrin-binding protein in its outer membrane under iron-limited conditions [57,58].

**Figure 2:**
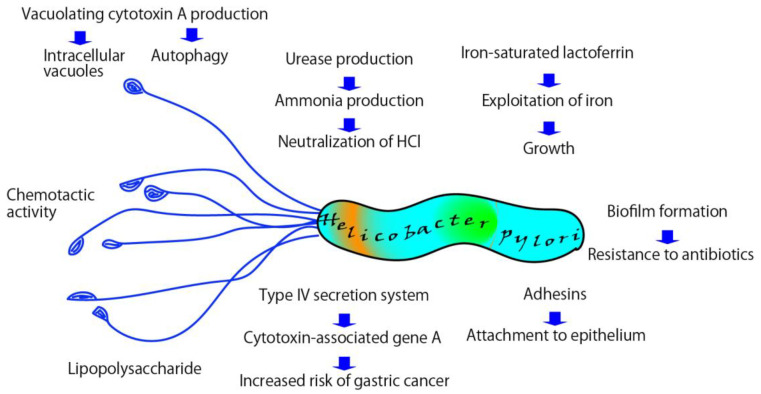
Survival strategy of *H. pylori* in the gastric mucosa. *H. pylori* harnesses several mechanisms, including the exploitation of iron, the expression of virulence factors, biofilm formation, and urease activity to survive in the gastric mucosa.

Other important factors for *H. pylori* colonization of the gastric mucosa include urease production, chemotactic motility, adhesins, biofilm formation, and virulence factors such as VacA and CagA. (Figure 2) [59,60]. VacA induces autophagy, autophagosomes, and the formation of intracellular vacuoles in host epithelial cells, thus enabling *H. pylori* colonization and survival in the gastric mucosa [59]. CagA, encoded by the cag pathogenic island (cag PAI) and linked to a type IV secretion system (T4SS), is associated with an increased risk of gastric cancer and peptic ulcer disease [61]. Rieder et al. reported that an intact T4SS enables *H. pylori* colonization of the gastric corpus in the Mongolian gerbil model [62]. Although the effects of LF on *H. pylori* T4SS in the stomach are not well understood, Lu et al. reported that apo-LF exerts antimicrobial activity against *H. pylori* under iron-limited conditions and that holo-LF suppresses cag T4SS activity [48]. Biofilm formation has been suggested to play a role in bacterial colonization and may be associated with antibiotic treatment failure [60]. Furthermore, iron is an essential nutrient for biofilm development and growth [63]. The anti-biofilm effects of LF in *H. pylori* infection are not yet fully understood, but in vitro studies using *P. aeruginosa* have demonstrated that LF inhibits biofilm formation [64].

As described above, LF has been shown to inhibit the growth of *H. pylori* in vitro through various mechanisms. However, *H. pylori* may preferentially take up iron from human LF through a species-specific LF-binding protein produced by *H. pylori*, thus enabling the bacterium to inhabit the stomach [57].

## 4. Antimicrobial Effect of LF against *H. pylori* In Vitro

LF has an exceptionally potent iron-binding ability, 260 times stronger than that of transferrin [65]. Thus, LF can effectively inhibit the growth of bacteria by depriving them of iron, an essential nutrient for their survival. Apo-LF (an iron-free molecule) can be microbiostatic due to its ability to sequester ferric iron, thus blocking the availability of host iron to pathogens, while holo-LF (the iron-saturated molecule) may provide iron to bacteria (Figure 1). Generally, LF purified from human and bovine milk has an iron saturation of 10–30%. Miehike et al. reported the direct activity of recombinant h-LF (rh-LF) against *H. pylori* in 1996 [66]. Thirteen clinical isolates of *H. pylori* were inoculated onto Brain Heart Infusion Agar supplemented with 7% fresh horse blood and incubated under microaerobic conditions. Human LF exerted a time- and dose-dependent action at a 1.5 mg/mL concentration against 8 of the 13 *H. pylori* isolates tested in vitro. In addition, we reported the antibacterial effects of LF and lactoferrin-derived peptide (LFcin, induced by pepsin digestion) against *H. pylori* in vitro [32]. The antibacterial activity of h-LF, b-LF, and LFcin against *H. pylori* was investigated using clinical isolates and a standard strain (ATCC43504). Bovine LF and h-LF at concentrations of 1.25–2.50 mg/mL completely inhibited the growth of *H. pylori* in Brucella broth, while holo-LF did not deter the growth of *H. pylori*. On the other hand, although bovine LFcin had little effect on the growth of *H. pylori* in Brucella broth, it inhibited it at concentrations of 0.1–1.0 mg/mL within 1 h of incubation in 1% peptone broth. Moreover, bovine LFcin inhibited the urease activity, the main virulence factor for *H. pylori* to colonize the gastric mucosa. However, the sensitivity of *H. pylori* to LFcin varied among the strains tested. Similarly, Dial et al. investigated the effect of b-LF on *H. pylori* in vitro and reported that b-LF was bacteriostatic to *H. pylori* when cultured at a concentration of ≥0.5 mg/mL, whereas another milk constituent, lysozyme or LFcin B, did not inhibit the growth of *H. pylori* [67]. In summary, apo-LF has antibacterial activity against *H. pylori* in vitro.

## 5. Antimicrobial Effects of LF in Animal Models

Wada et al. investigated the effects of b-LF administration on germ-free BALB/c mice [68]. Three weeks post-infection, the mice were administered b-LF orally once daily for two or four weeks, after which they were euthanized to evaluate the bacterial count in the stomach and the serum anti-*H. pylori* antibody titer. The administration of b-LF for three to four weeks reduced the number of *H. pylori* in the stomach, and the serum antibody titer decreased to an undetectable level. Dial et al. studied the in vivo effects of recombinant h-LF (rh-LF) on mice infected with *H. felis* [69]. The two-week treatment with rh-LF was sufficient to partially improve both *H. felis*-induced gastritis and the infection rate. Thus, they advocated further testing this promising agent for *H. pylori* eradication therapy. Conversely, Huynh et al. conducted a prevention and a treatment trial of b-LF and rh-LF on *H. pylori* infection in female C57BL/6 mice and found that b-LF and rh-LF were unable to reduce *H. pylori* load, with gastric myeloperoxidase (MPO) activities being higher with LF treatment [70].

## 6. Effect of b-LF on Urease Activity in Humans

Our previous study showed that b-LF and b-LFcin had antimicrobial activity against *H. pylori* in vitro, but the effect of LF in animal models was not bactericidal [32,68,69,70]. Therefore, we examined the impact of orally administered b-LF on *H. pylori* infection in humans [30]. The participants were 24 volunteers with *H. pylori* infection confirmed by the ^13^C-urea breath test (UBT). Fifteen volunteers received yogurt containing 0.4 g of LF daily for eight weeks (LF group), while the other nine received yogurt without LF (control group). The infection status of *H. pylori* was assessed using the UBT at four-week intervals. In the LF group, the UBT value (mean ± standard deviation; per mille) was significantly decreased after 4 (31.3 ± 15.4‰; *p* = 0.0192) and 8 (24.2 ± 11.9‰; *p* = 0.0016) weeks of treatment compared to the baseline value (43.0 ± 28.2‰). In the control group, the UBT value was 32.2 ± 17.7‰ before treatment, 27.2 ± 11.2‰ after four weeks, and 30.3 ± 15.9‰ after eight weeks. The UBT value remained relatively unchanged over the 8-week study period. Despite the significant decrease in the UBT value observed in the LF group, likely due to both *Lactobacillus* and *Bifidobacterium* in yogurt, the current results may be attributed to the combined effects of LF and probiotics. Probiotics may have a suppressive impact on *H. pylori* infection [71]. Therefore, Okuda et al. conducted a randomized, double-blind, placebo-controlled study to evaluate the single effect of orally administered b-LF against *H. pylori* (Table 1) [31]. Fifty-nine healthy individuals with *H. pylori* infection were randomly assigned to two groups. The b-LF group received b-LF tablets at a dosage of 200 mg b.i.d. for 12 weeks, and the control group was given placebo tablets without b-LF. The urease activity of *H. pylori* infection was assessed by UBT at baseline, during and after the administration period, and again four weeks post-administration. A positive response was defined as a decrease of more than 50% in the UBT value at the end of the administration. Ten out of the thirty-one b-LF-treated subjects (32.3%) exhibited a positive response, compared to only one out of the twenty-eight control subjects (3.6%), indicating that the rate of positive response in the b-LF group was significantly higher than that in the control group (b-LF vs. control, *p* < 0.01). These results suggested that the oral administration of b-LF effectively suppressed the urease activity of *H. pylori*; however, the UBT values returned to the baseline levels four weeks after the end of b-LF administration. Therefore, a single oral use of b-LF can reduce the urease activity of *H. pylori* but does not appear to be bactericidal against *H. pylori* in humans.

## 7. The Effect of b-LF Supplementation on *H. pylori* Eradication Therapy

Following a single oral dose of b-LF in humans, urease activity is inhibited, but the therapy does not eradicate *H. pylori* [31]. Therefore, b-LF supplementation in combination with empiric triple therapy has been explored as a potential treatment for *H. pylori* eradication. In 2003, Di Mario et al. conducted a preliminary study that revealed that b-LF in combination with a standard triple therapy regimen of rabeprazole, clarithromycin, and tinidazole for seven days led to a significantly higher eradication rate (100%; 24/24) than the standard triple therapy for seven days (76.9%; 20/26, *p* = 0.023) and ten days (70.8%; 17/24, *p* = 0.022) (Table 1) [72]. Furthermore, they highlighted the good patient compliance with the treatment schedule and the relatively low cost of LF in this quadruple therapy. Subsequently, Di Mario and his colleagues conducted another open, randomized, single-center study with the same regimens, including 150 consecutive *H. pylori*-positive patients [73]. The results showed that the 7-day quadruple therapy group with LF reported a high eradication rate (intention-to-treat (ITT)/per protocol (PP); 92.2/95.9%) compared to the standard triple therapy for seven days group (71.2/72.5%) and the ten days group (70.2/75%). The quadruple therapy with LF led to significantly higher eradication rates than the other two regimens (*p* = 0.01; ITT analysis, *p* = 0.005; PP analysis). Furthermore, they conducted an open, randomized, multicenter, prospective study with 402 patients, who were divided into three regimens: Group A received esomeprazole, 20 mg twice daily, clarithromycin, 500 mg twice daily, and tinidazole, 500 mg twice daily for seven days; Group B received LF, 200 mg twice daily for seven days followed by the same regimen as Group A; and Group C received the concurrent administration of LF, 200 mg twice daily, with the same regimen as Group A. In this study, the eradication rates (ITT) were 77% (105/136) in Group A, 73% (97/132) in Group B, and 90% (120/134) in Group C, with a statistically significant difference between the groups (Chi-square test; *p* < 0.01). The incidence of side effects did not vary significantly among the three treatment groups [74].

Similarly, Tursi et al. conducted a prospective, randomized study on 70 consecutive patients who had failed to respond to the standard first-line therapy (Table 1) [75]. All patients were randomly assigned to one of two groups: Group A, which received ranitidine bismuth citrate (RBS, 400 mg b.i.d.), esomeprazole (400 mg/day), amoxicillin (1 g t.i.d.), and tinidazole (500 mg b.i.d.), and Group B, which received the same treatment plus b-LF supplementation (200 mg b.i.d.). As a result, 67 patients completed the study, and the *H. pylori* eradication rate was 88.57% (ITT, 95%CI; 87-99%) in Group A and 94.28% (ITT 95%CI; 86-100%) in Group B. Although the cure rate of *H. pylori* showed no significant difference between both groups, the incidence of side effects was significantly lower in Group B (29.41% vs.17.64%; *p* = 0.05). Therefore, they concluded that LF supplementation effectively reduced the incidence of side effects.

De Bortoli et al. investigated whether LF and probiotics could enhance the efficacy of the standard triple therapy (esomeprazole, clarithromycin, and amoxicillin) (Table 1) [76]. The patients were randomized into two groups: Group A without LF plus probiotics supplementation and Group B with LF plus probiotics supplementation. According to the ITT analysis, 72.2% of the patients in Group A and 92.1% of the patients in Group B were cured of the infection. The PP analysis showed that 76.0% of the patients in Group A and 92.1% of the patients in Group B were successfully treated. Furthermore, the side effects were significantly lower in Group B than in Group A (*p* < 0.05). Therefore, they concluded that adding b-LF and probiotics could enhance the efficacy of the standard therapy and reduce the side effects of combined antibiotics.

Conversely, Zullo et al. reported different results in their 2005 prospective, open-label, randomized, multicenter trial involving 133 consecutive patients [77]. The patients were divided into two groups: Group A, who received esomeprazole, 20 mg twice daily, clarithromycin, 500 mg twice daily, and amoxicillin, 1g twice daily for seven days; and Group B, who received the same regimen plus LF 200 mg twice daily for seven days. The *H. pylori* eradication rate was 77.9% (53/68) in Group A and 80.3% (53/66) in Group B. No significant difference was found between the two groups, and the effect of adding LF was not confirmed. Furthermore, they compared a 7-day quadruple therapy, including rabeprazole, clarithromycin, tinidazole, and LF, with a levofloxacin-based triple therapy (rabeprazole, levofloxacin, and amoxicillin). However, in this trial, the LF group included clarithromycin with a high resistance rate against *H. pylori*, while the levofloxacin group included amoxicillin with a low resistance rate [78]. It is, therefore, difficult to draw any conclusions regarding the additional efficacy of LF in this study.

In 2009, two research groups conducted a meta-analysis to determine whether LF has an add-on effect on the standard triple therapy (Table 1) [79,80]. Zou et al. analyzed nine randomized trials (n = 1343) and found that the patients with LF supplementation reported an eradication rate of 86.57%, compared to 74.44% in those without LF supplementation [79]. Furthermore, the total incidence of side effects was 9.05% and 16.28% for the groups with and without LF, respectively. Therefore, they concluded that the supplementation of LF could effectively increase the cure rates of anti-*H. pylori* therapy and could be beneficial for patients undergoing the eradication therapy [79]. Similarly, Sachdeva and Nagpal identified five eligible randomized clinical trials (RCTs) (of 169) with 682 subjects (b-LF group-n = 316; control group-n = 366) [78]. The pooled odds ratio (five studies) for eradication by ITT was 2.22 (95% CI 1.44–3.44; *p* = 0.0003) using the fixed effects model (FEM) and 2.24 (95% CI 1.15–4.35; *p* = 0.0003) using the random effects model (REM) (Cochran’s Q = 6.83; *p* = 0.145). Thus, they concluded that b-LF might improve *H. pylori* eradication rates without any increase in adverse effects [80]. Furthermore, the recent report by Hablass et al. in 2021 demonstrated the same efficacy of LF supplementation in the PPI-based triple therapy or sequential therapy for *H. pylori* eradication [81]. In summary, although there are some conflicting results, the supplementation of b-LF may have an add-on effect on the proton pump inhibitor-based triple therapy for *H. pylori* eradication [77].

## 8. Conclusions

We reviewed the antimicrobial effects of b-LF on *H. pylori* infection both in vitro and in vivo and discussed the potential efficacy of b-LF supplementation in the triple therapy for *H. pylori* infection. *H. pylori* is a unique bacterium adapted to the human gastric mucosa. *H. pylori* can evade the antimicrobial effect of and exploit the iron bound to h-LF. However, the oral administration of b-LF is not effective enough to completely eliminate *H. pylori* in clinical practice. Therefore, b-LF has been combined with the conventional triple therapy for *H. pylori* eradication. The limitation of this review is that most of the studies with b-LF supplementation were conducted mainly by two groups in Italy. B-LF is commercially available mixed in yogurt and is cost-effective in Japan. B-LF-based regimens may be recommended after previous *H. pylori* eradication failure.

## Figures and Tables

**Table 1:** Clinical trials with or without lactoferrin or standard therapy with or without lactoferrin.

Subjects	Study Design	Study Groups	Results	References
59 volunteers	Randomized controlled trial	(1) b-LF-treated group; (2) placebo-treated control group	Suppression of *H. pylori* colonization	Okuda et al., 2005 [31]
150 patients	Open randomized single-center study (preliminary results)	(1) Triple therapy (rabeprazole, clarithromycin, tinidazole) + LF for 7 days; (2) triple therapy (rabeprazole, clarithromycin, tinidazole) for 7 days; (3) triple therapy (rabeprazole, clarithromycin, tinidazole) for 10 days	Significantly higher eradication rate in triple therapy + LF than other groups	Di Mario et al., 2003 [72]
150 patients	Open randomized single-center study	(1) Triple therapy (rabeprazole, clarithromycin, tinidazole) + LF for 7 days; (2) triple therapy (rabeprazole, clarithromycin, tinidazole) for 7 days; (3) triple therapy (rabeprazole, clarithromycin, tinidazole) for 10 days	Significantly higher eradication rate in triple therapy + LF than other groups	Di Mario et al., 2003 [73]
402 patients	Open, randomized, multicenter, prospective study	(1) Triple therapy (esomeprazole, clarithromycin, tinidazole) for 7 days; (2) b-LF for 7 days followed by triple therapy (esomeprazole, clarithromycin, tinidazole) for 7 days; (3) triple therapy (rabeprazole, clarithromycin, tinidazole) + b-LF for 7 days	The eradication rate was significantly higher in patients receiving b-LF	Di Mario et al., 2006 [74]
70 patients	Prospective randomized clinical trial after failure of first standard treatment	(1) Ranitidine bismuth citrate, esomeprazole, amoxycillin, tinidazole; (2) ranitidine bismuth citrate, esomeprazole, amoxycillin, tinidazole + b-LF	The group receiving b-LF showed a higher but not statistically significant eradication rate.	Tursi et al., 2007 [75]
206 patients	Prospective randomized study	(1) Triple therapy (esomeprazole, clarithromycin, tinidazole); (2) triple therapy (esomeprazole, clarithromycin, tinidazole) + b-LF + probiotics	The eradication rate was 92.1% in the group receiving triple therapy + b-LF + probiotics and 76% in the group receiving only the standard triple therapy	De Bortoli et al., 2007 [76]
133 patients	Prospective, open l-label, three-center, randomized study	(1) Triple therapy (esomeprazole, clarithromycin, amoxycillin); (2) triple therapy (esomeprazole, clarithromycin, tinidazole) + bLF for 7 days	The eradication rate was 80.3% in the group receiving triple therapy + b-LF and 77.9% in the group receiving only the standard triple therapy. No significant difference between groups	Zullo et al., 2005 [77]
144 patients	Prospective, open l-label, multicenter, randomized study	(1) Triple therapy (rabeprazole, levofloxacin, amoxycillin) for 7 days; (2) triple therapy (esomeprazole, clarithromycin, tinidazole) + b-LF for 7 days	The eradication rate was 69.1% (per protocol analysis) in the group receiving triple therapy and 76.5% in the group receiving quadruple therapy	Zullo et al., 2007 [78]
9 randomized clinical trials (n = 1343 subjects)	Meta-analysis	(1) Triple therapy (proton-pump inhibitor + 2 antibiotics) or quadruple therapy (proton-pump inhibitor + bismuth + 2 antibiotics; or ranitidine bismuth citrate + same antibiotics); (2) b-LF-including regimens	The eradication rate was 86.57% in the group receiving standard therapy + b-LF and 74.44% in the group receiving only standard therapy	Zou et al., 2009 [79]
5 randomized clinical trials (n = 682 subjects)	Meta-analysis	(1) Standard therapy; (2) standard therapy + b-LF	The pooled odds ratio by intention-to-treat analysis in the b-LF vs. non-b-LF group was 2.22 and 2.24 using the fixed effects model and the random effects model, respectively	Sachdeva et al., 2009 [80]
400 patients	Randomized controlled clinical trial	(1) Proton pump inhibitor-based triple therapy for 2 weeks; (2) sequential therapy for 2 weeks; (3) proton-pump-based triple therapy + b-LF for 2 weeks; (4) sequential therapy + b-LF for 2 weeks	The success rates were 70.3%, 82.8%, 85.6%, and 94.5% in groups (1), (2), (3), and (4), respectively	Hablass et al., 2020 [81]

## Data Availability

Not applicable.
